# Bio-Inspired Hierarchical Micro/Nanostructured Surfaces for Superhydrophobic and Anti-Ice Applications

**DOI:** 10.3389/fbioe.2022.872268

**Published:** 2022-03-21

**Authors:** Lansheng Zhang, Paul C. Uzoma, Chu Xiaoyang, Oleksiy V. Penkov, Huan Hu

**Affiliations:** ^1^ ZJU-UIUC Institute, International Campus, Zhejiang University, Haining, China; ^2^ State Key Laboratory of Fluidic Power and Mechanical Systems, Zhejiang University, Hangzhou, China

**Keywords:** hierarchical micro/nanostructures, superhydrophobic, anti-icing, GLAD, deep reactive ion etching

## Abstract

We report a scalable and cost-effective fabrication approach for constructing bio-inspired micro/nanostructured surfaces. It involves silicon microstructure etching using a deep reactive ion etch (DRIE) method, nanowires deposition *via* glancing angle deposition (GLAD) process, and fluorocarbon thin film deposition. Compared with the smooth, microstructured, and nanostructured surfaces, the hierarchical micro/nanostructured surfaces obtained *via* this method showed the highest water contact angle of ∼161° and a low sliding angle of <10°. It also offered long ice delay times of 2313 s and 1658 s at −5°C and −10°C respectively, more than 10 times longer than smooth surfaces indicating excellent anti-icing properties and offering promising applications in low-temperature environments. These analyses further proved that the surface structures have a significant influence on surface wettability and anti-icing behavior. Hence, the GLAD process which is versatile and cost-effective offers the freedom of constructing nanostructures on top of microstructures to achieve the required objective in the fabrication of micro/nanostructured surfaces when compared to other fabrication techniques.

## Introduction

The formation of ice on artificial surfaces causes significant problems to industries and human life qualities. Ice accretion on the wings of an aircraft’s cab alters the aerodynamic characteristic of aircraft leading to flight stability and safety issues (Cao et al., 2015); icing on blades of wind turbines causes reduced energy generation efficiencies, degradation of the aerodynamic performance, and even flight accidents (Kraj and Bibeau, 2010; Dong et al., 2020); icing on thermal exchanger surfaces such as those in refrigerators increases the thermal resistance between the refrigerant and the surrounding air thus reduce the heat exchange efficiency (Malik et al., 2020); icing on walls of tall buildings and towers pose a serious threat to people and properties on the ground (Carter and Stangl, 2012); ice stack on the antenna, camera, solar panels also lead to a drop in output efficiency and durability (Rahmatmand et al., 2018). These challenges and more are behind the drive to design and construct durable icephobic surfaces to suppress icing and protect surfaces from being covered with ice.

Existing solutions to icing problems fall into active and passive categories. Active approaches use external energy sources such as heating wires, hot airs, mechanical vibration, or ultrasonic to remove ice (Tian et al., 2015; Gao et al., 2019; Liu et al., 2020a; Shan et al., 2020), which are efficient but costly, require complex piping systems, and is demanding high energy. Passive approaches use a combination of physical and chemical methods such as slippery liquid-infused porous surface (SLIPS), coating, electrochemical deposition, etching, self-assembly technique, etc. (Kumar et al., 2019; Latthe et al., 2019). Passive approaches are preferred in situations where human operation is difficult like in tall buildings, power towers, etc. In passive approaches, adding a layer of icephobic coating is the most common method. For example, Wu et al. prepared an anti-icing coating on concrete using fluorinated silicon-based copolymer adhesive and nano-silica (Wu et al., 2020). Paul et al. reported the use of functionalized nanodiamonds and acrylic resin to design an icephobic coating on an aluminum substrate (Uzoma et al., 2021). Also, Huang et al. has proposed the use of hydrogels to design anti-icing coatings on various kinds of substrates (Huang et al., 2020). Interestingly, most of the reported icephobic coatings also exhibit superhydrophobic properties. However, the anti-icing ability by coating a substrate with a thin superhydrophobic film is not sufficient to satisfy the end-use requirements because they easily fail under the conditions of mechanical abrasion, high humidity, and heavy snowfall (Kreder et al., 2016; Latthe et al., 2019).

Inspired by Nature, scientists have designed superhydrophobic surfaces for many crucial applications such as anti-icing (Onda et al., 1996; Lin et al., 2011; Latthe et al., 2019), biosensing (Xu et al., 2017; He et al., 2018; Song et al., 2018; Xu et al., 2018; Xu et al., 2019), energy storage (Sun et al., 2019), and surface-enhanced Raman scattering (SERS) (Chen et al., 2018). The hierarchically structured surface was found to be one of the key factors for the superhydrophobic property because it provides a composite surface consisting of both solid surfaces as well as air pockets, thereby increasing the contact angle and rendering more repellent surfaces for water (Feng et al., 2002; Liu et al., 2020b; Wu et al., 2021). Hence, water droplet from rains or snow, or condensed water droplet can roll off the surface before it forms ice.

Wenzel and Cassie’s models have been used to effectively describe the behavior of droplets on a surface (Wenzel, 1936; Cassie and Baxter, 1944). A droplet on a solid surface can either spread or contract till the contact angle with the surface gets to a certain value. The value of the angle is known to depend on the parity between the interfacial contact to reduce the surface free energy. On a rough surface, Wenzel proved that there is an increase in the contributions of the solid-liquid and solid-vapor areas to the surface free energy. His equation also suggests that the surface texture affects the essential wetness behavior because it assumes that the liquid remains in contact with the solid surface at all points within the projected droplet’s contact area. Hence, in Wenzel’s state, the water will fill into the micro/nanostructure of a textured surface. On the other hand, Cassie described a situation where the drop is suspended by air pockets trapped inside the textured surface suggesting that the liquid-gas interface replaced some of the liquid-solid interface (Cassie and Baxter, 1944; Uzoma et al., 2019). Within the structured surface approach, hierarchical micro/nanostructured (MN) surfaces achieve larger contact angles and smaller sliding angles for water droplets therefore potentially enhancing anti-icing properties (Guo et al., 2012; Guo et al., 2014). Shirtcliffe et al. have shown an increase in the superhydrophobic properties of hierarchical MN surfaces when the microstructure is in the Wenzel state and the nanostructure is in the Cassie state (Shirtcliffe et al., 2004). They suggested that for surfaces with dual-length scale roughness, the upward part of the surface tension of a water drop suspended between two short pillars could contribute to the influence of smaller scale roughness at the base of the pillars permitting the suspension of the water drop on the smaller scale roughness. This will make a micro/nanostructured surface show a large water contact angle while possessing relatively lower surface roughness. Also combining a rough base with smooth pillars can protect the rough surface against wear. Peng and co-workers demonstrated that MN surfaces can impact durable anti-icing property more than surfaces with only nanostructure, microstructure, or smooth surface (Guo et al., 2012). Bhushan et al. proved that hierarchical MN surfaces can overcome scale-dependent contact angle hence creating stable superhydrophobic states (Michael and Bhushan, 2007). (Wang et al., 2022) recently design MN surfaces that can spontaneously transition from Wenzel to Cassie state during the icing/deicing cycle. The surface was fabricated using the ultrafast laser ablation method.

MN surfaces are not straightforward to produce because a single fabrication approach is difficult to produce both microstructures and nanostructures. Du et al. used a combination of laser interference lithography, reactive ion etching, and e-beam deposition techniques to fabricate MN surface (nanoporous trilayer composite films) (Du et al., 2013). Electron beam lithography was applied to fabricate hierarchical micro/nanostructures but suffers from high cost and small area (Feng et al., 2011; Pattantyus-Abraham et al., 2013; Kumar et al., 2019). Normally, fabricating these types of surfaces requires a combination of a microfabrication process and a nanofabrication process. For example, microfabrication consisting of a UV lithography and etching as well as metal-assisted chemical etching was used to produce monolithic silicon hierarchical MN surfaces (Hu et al., 2014), but this method is only applicable to single-crystal silicon. Others use CVD-grown ZnO nanowires on top of microstructures, but normally require high temperature and furnaces to prepare (Bhujel et al., 2019; Choi et al., 2021). The machining process has also been employed to produce micro-rachets together with nano hairs prepared by crystal growth (Guo et al., 2012).

Here, we report a new process of producing hierarchical MN surfaces with a large contact angle of ∼161° and a small sliding angle of ˂10°. Moreover, we demonstrated a long icing delay time (IDT) of 1658 s and 2313 s at −5°C and −10°C respectively, both are more than 10 times longer than the IDT on smooth surfaces without MN structures. Furthermore, we showed the fabrication of MN surfaces on a 4-inch wafer scale in a low-cost fashion, which is very promising in practical applications.

## Experiment

### Fabrication Process

**FIGURE 1:**
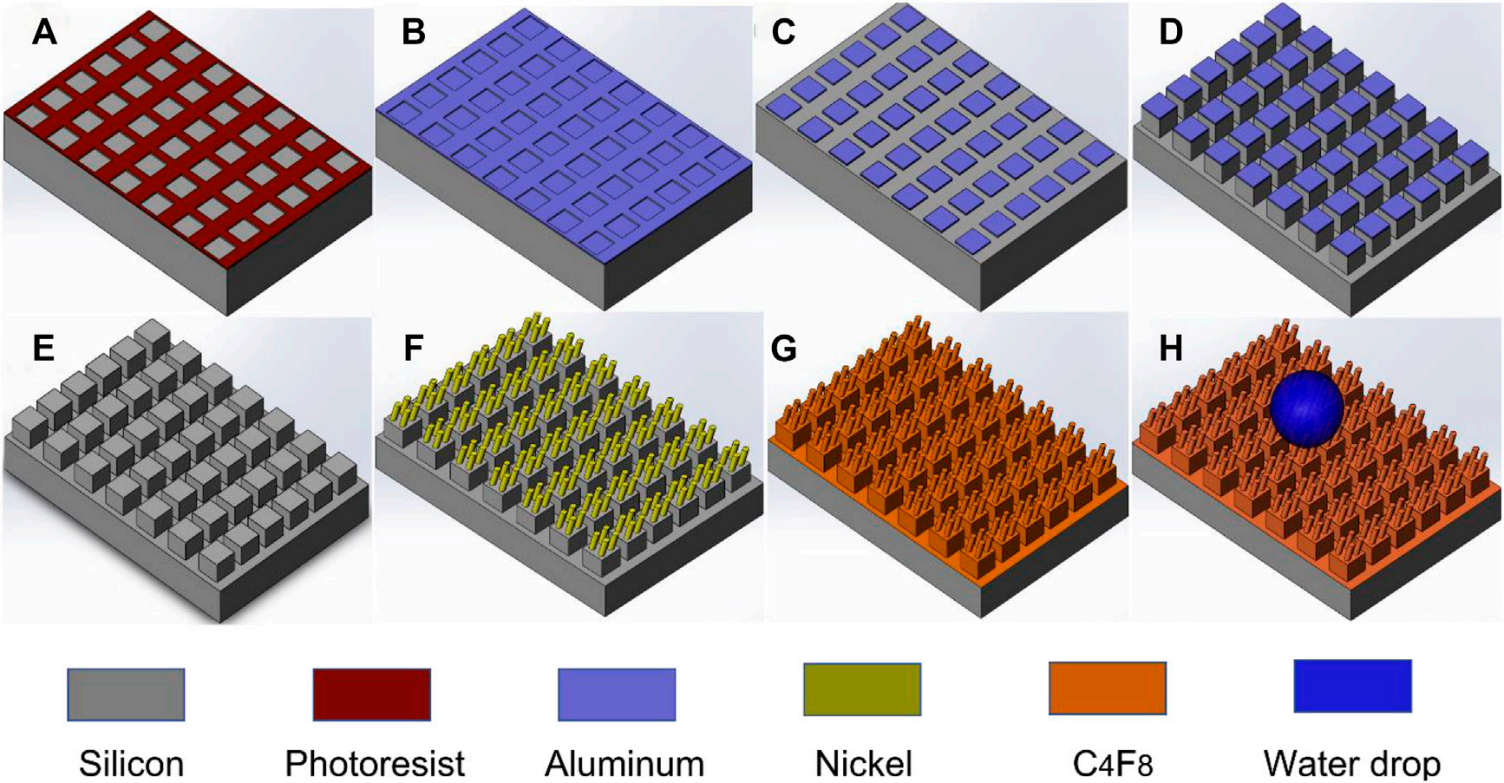
Schematic illustration of the major fabrication processes of micro/nano hierarchical structure. **(A)** UV photolithography; **(B)** Aluminum deposition; **(C)** Aluminum lift-off process; **(D)** Deep reactive ion etch; **(E)** Removal of aluminum; **(F)** GLAD nickel nanowires; **(G)** C_4_F_8_ deposition; **(H)** A water drop on the surface.

The fabrication of the hierarchical micro/nanostructured surface involves three main processes: silicon microstructure fabrication, nanowires deposition, and fluorocarbon C_4_F_8_ thin film deposition as described in Figure 1. The substrate used is a p-type <100> silicon. A micro-pattern of photoresist was designed *via* UV photolithography (step a), followed by the deposition of 300 nm thick aluminum (step b), and thereafter, applied the lift-off process to produce an aluminum metal mask (step c). Deep reactive ion etch (DRIE) was employed to etch silicon using the aluminum pattern as a mask (step d). Then aluminum etchants were used to etch aluminum to produce micro silicon pillar arrays (step e). Following these steps, the GLAD process was used to produce nickel nanowires on the micropillar’s surface (step f). Finally, 20 nm thick amorphous fluorocarbon film was deposited on the surface to reduce the surface energy and improve water resistance (step g). Step h shows a schematic diagram of water drop on the hierarchical micro/nanostructured surface.

### Glancing Angle Deposition Process

The nickel nanowires (step f) were fabricated using a custom-designed electron-beam evaporation system. The source materials for evaporation were nickel pellets (Ni 13,301, Ф: 3 mm × 3 mm, purity: 99.9%) obtained from Zhong Nuo Advanced Material Technology Co., Limited. The incident angle was fixed at 86° degrees to allow the self-formation of nanopillars, and the vacuum pressure was 4 × 10^–6^ Torr. The deposition rate was maintained at 0.2 nm/s. Nickel is used because of its good wear and corrosion resistance, and ductility which is particularly useful in anti-icing applications (Bassford et al., 1998).

**FIGURE 2:**
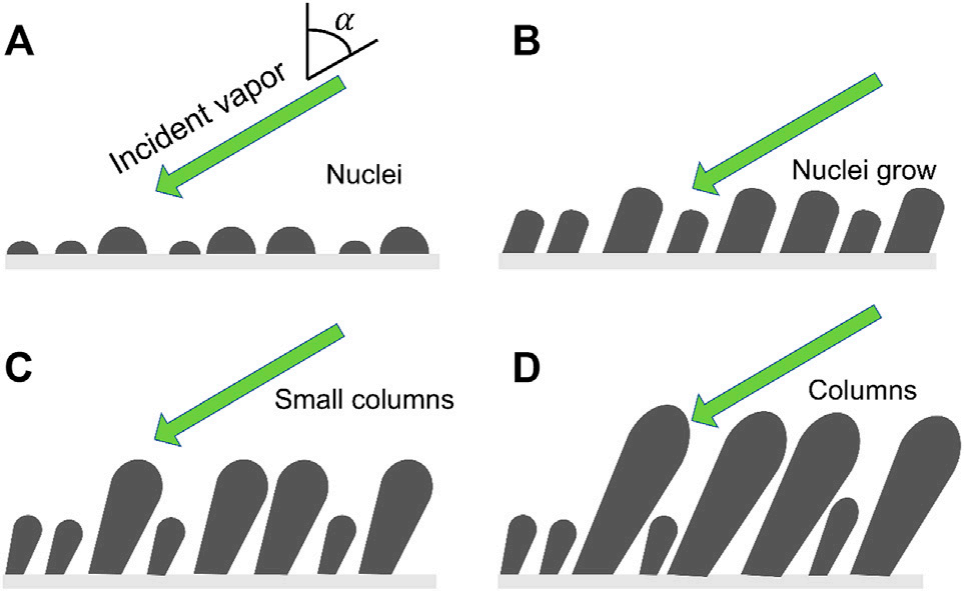
Schematic view of GLAD nanowires growth. **(A)** Vapor flux at an angle *α* produces a random distribution of nuclei on a substrate surface, **(B)** nuclei grow; **(C)** the nuclei develop into columns, some smaller columns were shadowed and stopped their growth; **(D)** columns growth and the further growth is restricted to the top of columns.

Figure 2 shows the growing process of nanowires using GLAD. It starts with the formation of the diverse sizes of the deposited random islands followed by the gradual amplification of the surface topography via ballistic shadowing. Hence a planar substrate will roughen through Volmer–Weber mode growth (Taschuk et al., 2010) and the resultant defects will also accelerate surface roughening. The initial stage of GLAD nanowires growth is shown in Figure 2A. The nuclei grow into columns and develop shadows as shown in Figure 2B. The columns and shadows screen the neighboring nuclei from incoming vapor flux thereby suppressing their growth (Figure 2C). The smaller nuclei and columns are completely shadowed and stop growing as seen in Figure 2D. Eventually, nanowires are formed without using nanotemplate or other expensive nanolithography techniques.

The key factors to have the nanowires successfully on surfaces are that the incident evaporation vapor is not blocked by other micropillars and the incident evaporation vapor has a certain angle with the surface similarly like with the top surfaces. When the evaporation vapor is almost vertical to the front sidewall surface as the normal evaporation, a thin film is formed instead of nanowires. When the sidewalls are not able to be touched by the evaporation source, no films or nanowires can be formed. When the sidewalls are almost parallel to the evaporation vapor forming a very small angle, nanowires are formed. Most areas on the bottom surfaces have nanowires except areas that are shadowed by micropillars. Supplementary Figure S1 in supplementary materials shows the surfaces on different sidewalls of micropillars as well as on the bottom surfaces. Supplementary Figure S2 illustrates the effects of the wafer size and position of the substrate on the heights of the nanowires.

The equilibrium contact angle (CA) is widely used to characterize the wetting behavior of a surface. The well-known Cassie–Baxter theory describes the equilibrium CA of a composite surface where vapor pockets are trapped underneath the liquid as expressed by the following equation (Cassie and Baxter, 1944):

$$cos\theta^{*}=f\left(cos\theta_{y}+1\right)-1$$ (1)

**FIGURE 3:**
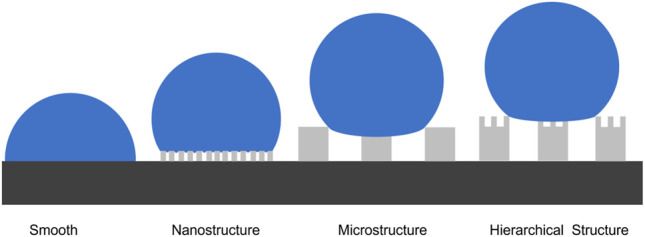
Schematic illustration of the water droplet on the differently structured surfaces.

Where θ^*^ represents the apparent CA. It is the sum of all the contributions of the liquid-solid and liquid-vapor interfaces as expressed in the Cassie-Baxter equation which is obtained using the contact angle goniometer and the ImageJ software., *f* is the area fraction of the solid that is in contact with the liquid, *θ*_y_ is the equilibrium CA of the liquid droplet on a smooth surface of the same substrate material. From this equation, we can adjust *f* and *θ*_y_ to increase the equilibrium CA. *f* is reduced by controlling the surface roughness and θ*y* is increased by the addition of low-surface-energy materials. In this paper, the surface roughness was increased by the fabrication of microstructure, nanostructure, and hierarchical structure (microstructure and nanostructure) on the surface and the reduction of the surface energy *via* the deposition of fluorocarbons (C_4_F_8_) on the surface. Figure 3 shows the schematic of the wetting behavior of a water drop on the differently structured surfaces. MN structures enable even smaller contact area of solid-liquid than only nanostructured or microstructured surfaces.

In order to determine the contributions of different surface structures to superhydrophobicity, we prepared four types of sample sets: hierarchical micro/nanostructured surface, microstructured surface, nanostructured surface, and smooth surface. These four surfaces were coated with an amorphous fluorocarbon (C_4_F_8_) film. The image of the droplet was taken by using a water drop shape imaging system and the water contact angle was measured by the ImageJ software. The sliding angle was measured by tilting the sample stage and recording when the droplet began to slide. All the droplets were generated by a micro-injector. Three duplicate measurements were taken for all the samples under normal laboratory ambient conditions.

### Anti-Icing Properties Measurements

Icing delay time (IDT) measurement was used to characterize the anti-icing ability of the four samples with different surface structures. The ice formation platform was designed using a Peltier thermoelectric generator sandwiched between a copper plate and a water-cooling unit as shown in Supplementary Figure S3. A digital temperature controller was attached to the platform to regulate the temperature. A 5 μL water droplet was used on a 1 cm × 1 cm sample area, and the time taken for the water droplet to turn into ice was recorded. When the ice was formed, the droplet lost its transparency easily as captured by image analysis. Three duplicate measurements were taken for all the samples at normal laboratory ambient conditions (22°C and 24% relative humidity).

## Results and Discussion

### Micro/nanostructured Surface Features

**FIGURE 4:**
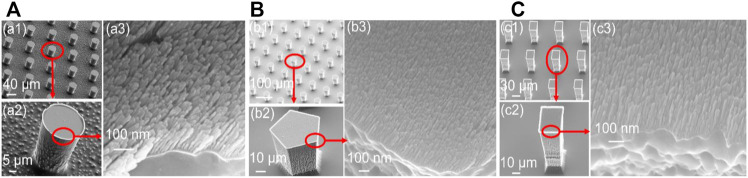
Three hierarchical micro/nanostructured surfaces with different microstructures, **(A)** cylinder, **(B)** regular pentagon columns, **(C)** rectangular columns

Figure 4 shows three different hierarchical micro/nanostructured surfaces from the above-described fabrication process in Figure 1 (steps a–h). As seen in Figure 4, images a, b, and c have three different silicon microstructures (cylinder, regular pentagon columns, and rectangular columns) fabricated by the DRIE process and the same nickel nanostructures fabricated by the GLAD process (a3, b3, c3). The dimensions of these three different microstructures are outlined in Table 1.

**TABLE 1:** Dimensions of three microstructures.

Microstructures	Diameter or length of side (μm)	Height (μm)	Unit distance (μm)
Cylinder	35.6	42	99
Regular pentagon columns	24.1	42	99
Rectangular columns	31.6	61	99

**FIGURE 5:**
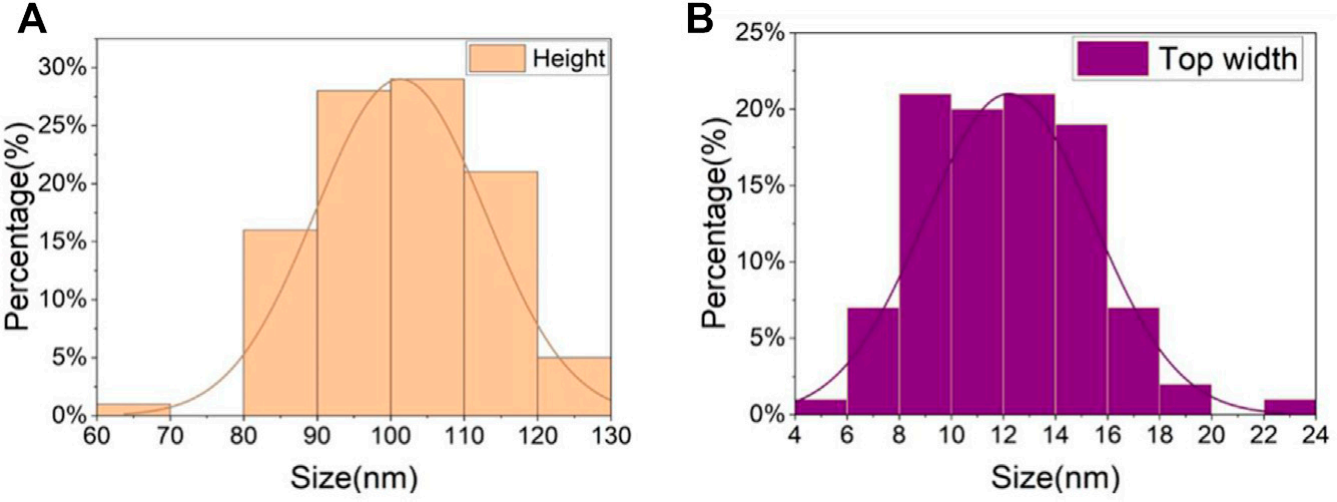
The measured statistic data of the nickel nanowire sizes. **(A)** height of nanowires, **(B)** top width of nickel nanowires.

Figure 4 (a3, b3, c3) shows the nickel nanowires produced using the GLAD process while Figure 5 describes the statistics of nanowires’ top width and height. 100 nanowires were measured in order. The average height of the nanowires is 101 nm with a higher concentration between 80 and 120 nm. The pillar top width concentrated between 10 and 14 nm with a 12 nm average. The evaporation time and metal thickness are used in controlling the height of the nanowires. Interestingly, the 3 MN structures in Figure 4 offered approximately the same CA value of 161° (a = 161.1 ± 0.5; b = 160.7 ± 0.8; c = 160.7 ± 0.6). This might be because the three structures possess the same unit distance as seen in Table 1 and the same area fraction as discussed in the next section. It suggests that the CA is less dependent on the shape of the microstructure.

### Wettability Analysis

**FIGURE 6:**
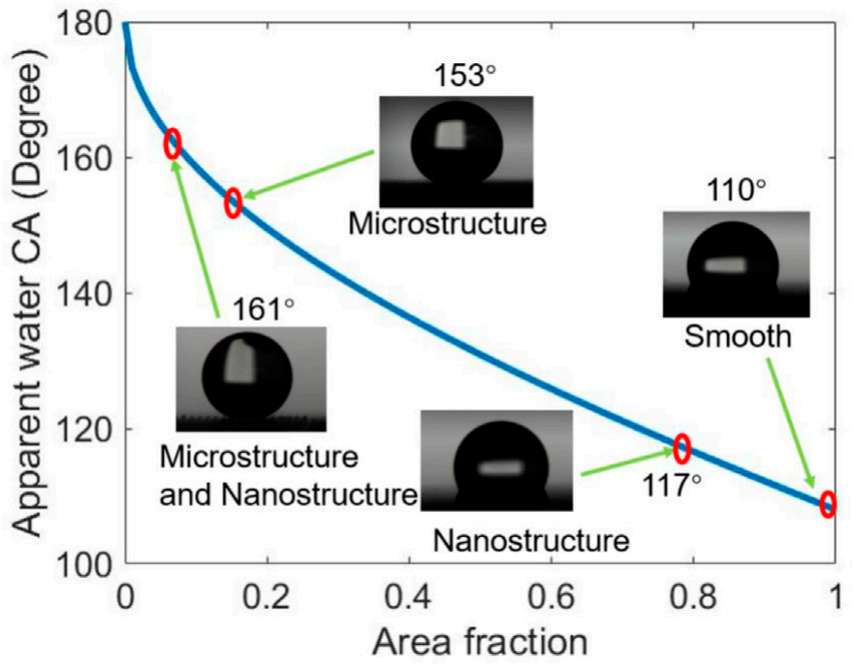
The equilibrium water contact angle of the various structured surfaces compared with the theoretical Cassie state curve. The apparent CA is the sum of all the contributions of the liquid-solid and liquid-vapor interfaces and is also the contact angle we measured using the goniometer and ImageJ software. And the area fraction is the fraction of the solid in contact with the liquid as described in the Cassie–Baxter equation.

Figure 6 shows the equilibrium water CA of the four different structured surfaces which are smooth (S), microstructured (M), nanostructured (N), and hierarchical micro/nanostructured (MN) surfaces. The obtained results are plotted on the theoretical Cassie state curve. The equilibrium water CA of the S surface with fluorocarbon film is 110° and the area fraction obtained using Eq. 1 is ∼1. The CA of the N surface is 117° and the same *f* value of 0.79 was obtained using Eq. 1 and Figure 4 (a3, b3, c3). This indicates that the nanopillars on the surface are close-knit, and there is a good agreement between the experimental and theoretical results. The equilibrium CA of the M surface is 153°, the experimented *f* value is 0.15, and the *f* value obtained from Figure 4 (a1, b1, c1) is 0.1 which is within the permitted error margin. For the MN surface, the equilibrium CA is 161° and the *f* value is 0.12 which is approximately close to the theoretical *f* value of 0.08. From the results, it is shown that the water droplet had a Wenzel wetting state contact with the S and N surfaces but showed a Cassie state contact with the M and MN surface. This can be attributed to the composite nature of the M and MN surfaces made of solid materials and trapped air. Also, the highest CA value seen in the MN surface is because of the hierarchical nature of the surface. These findings also prove that superhydrophobicity is largely dependent on multi-scale structures as seen in nature such as lotus leaves (Darmanin and Guittard, 2015; Neinhuis and Barthlott, 1997). Besides, the results are in concordance with literature; MN structured surfaces were shown both experimentally and theoretically that the presence of submicron and nanostructures can decrease the threshold of micropillar height to attain superhydrophobicity (Patankar, 2004; Sui et al., 2021). In cases of extremely small droplets, the nanostructures can prevent them from accessing the groves (Liu et al., 2011). The water contact angle hysteresis of M and MN surfaces are 9° and 15° respectively as shown in Supplementary Figure S4, indicating that the droplet is more likely to adhere to the M surface than the MN surface. Furthermore, Supplementary Video S1 proved that water droplets can easily roll off the MN surface at a very low tilt angle (< 10°). The low contact angle hysteresis and low sliding angle of the superhydrophobic surface are very essential for self-cleaning applications (Neinhuis and Barthlott, 1997; Yilbas et al., 2018). It is interesting to note that M structured surfaces have been reported to show good application potential (Song et al., 2020a; Song et al., 2020b; Fan et al., 2021).

### Anti-Icing Analysis

**FIGURE 7:**
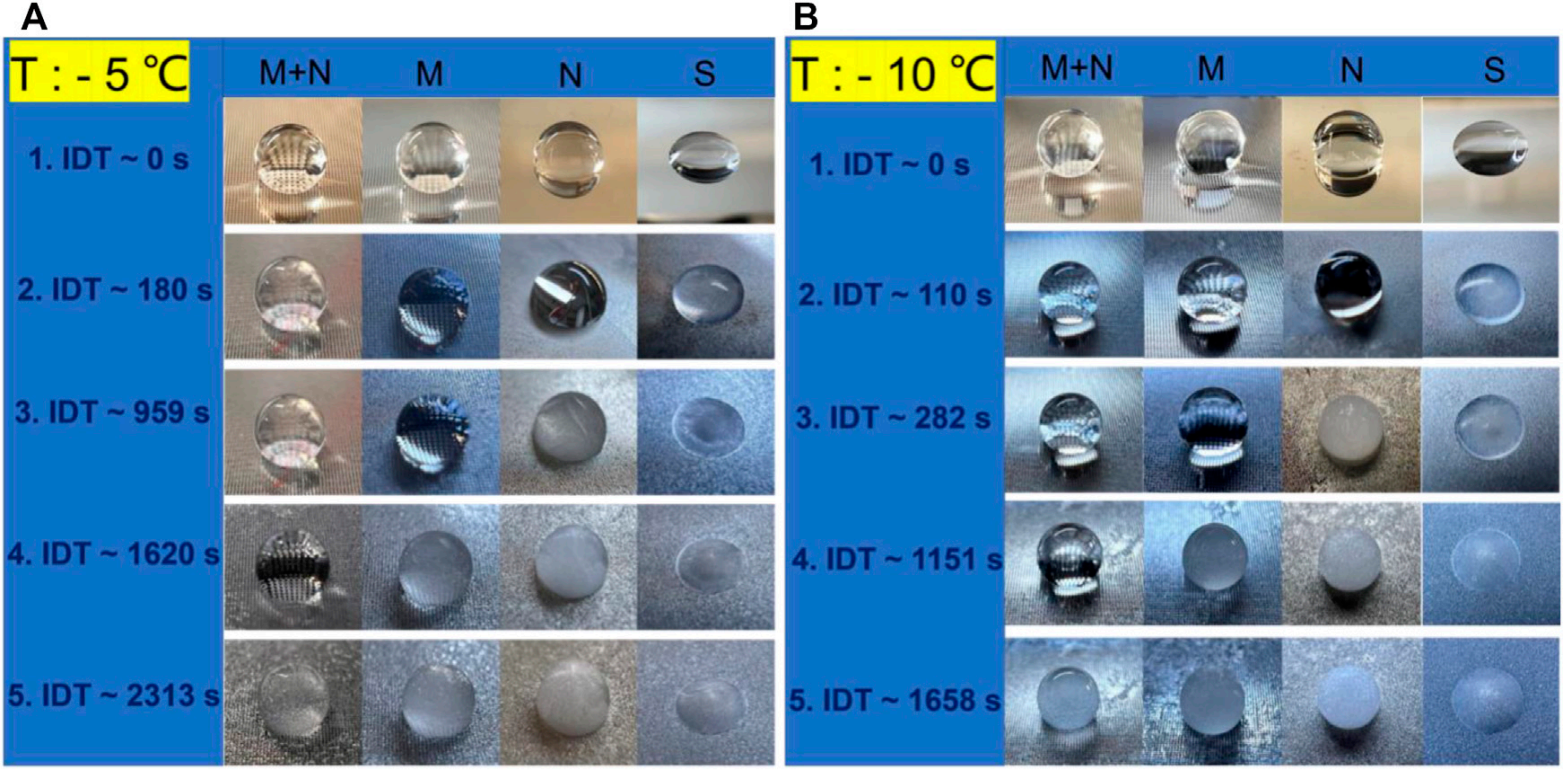
Observations of ice formation on the differently structured surfaces at −5°C and −10°C cooling temperatures. From right to left are the photos of the droplets on the surfaces of S, N, M, and MN, respectively. **(A)** Record of ice formation and IDT at −5°C: Frame 1 (IDT ∼0 s) shows the water drop on the surface prior to the experiment; Frame 2, the droplet on S surface freezes at ∼180 s IDT; Frame 3, the droplet on N surface freezes at ∼959 s; Frame 4, the droplet on M surface freezes at ∼ 620 s IDT; Frame 5, the droplet on MN freezes at ∼2313 s IDT. **(B)** Record of ice formation and IDT at −10°C with each frame showing a similar trend as **(A)**.

The icing delay time (IDT) of 5 μL water droplets on the S, N, M, and MN surfaces at −5°C and −10°C temperatures were measured, and the results are shown in Figure 7. The initial temperature of the droplet is 20°C, and the images in Figure 7 show the gradual transformation of the droplet from liquid to ice. The droplet on the S surface shows the least IDT of 180 and 110 s at −5°C and −10°C respectively, suggesting that normal smooth silicon surfaces do not have anti-icing properties. The icing times for the droplet on the N surface at −5°C and −10 °C are 959 and 282 s respectively, while the times to form ice for the droplet on the M surface at −5°C and −10°C are 1620 and 1151 s. The hierarchical MN surface offered the highest IDT of 2313 and 1658 s at −5°C and −10°C respectively, indicating that it has improved anti-icing properties compared to the other structured surfaces. Furthermore, the results showed a reduction in the IDT as the temperature decreases from −5°C to −10°C; the different surfaces S, N, M, and MN exhibited 39, 70, 29, and 28% respective decrease in the IDT values. This proves that the ice delay time is strongly influenced by the substrate’s temperature because decreasing the substrate’s temperature could alter the CA of the water droplet and eventually lead to the Cassie-Wenzel transition (Su et al., 2016; Li and Guo, 2018). MN surface is the least affected by the temperature change suggesting improved Cassie state stability. Besides, there are no observable changes in the SEM images of the MN surfaces after 10 cycles of icing/deicing as shown in Supplementary Figure S5 signifying that the ice formation did not destroy the MN surfaces.

**FIGURE 8:**
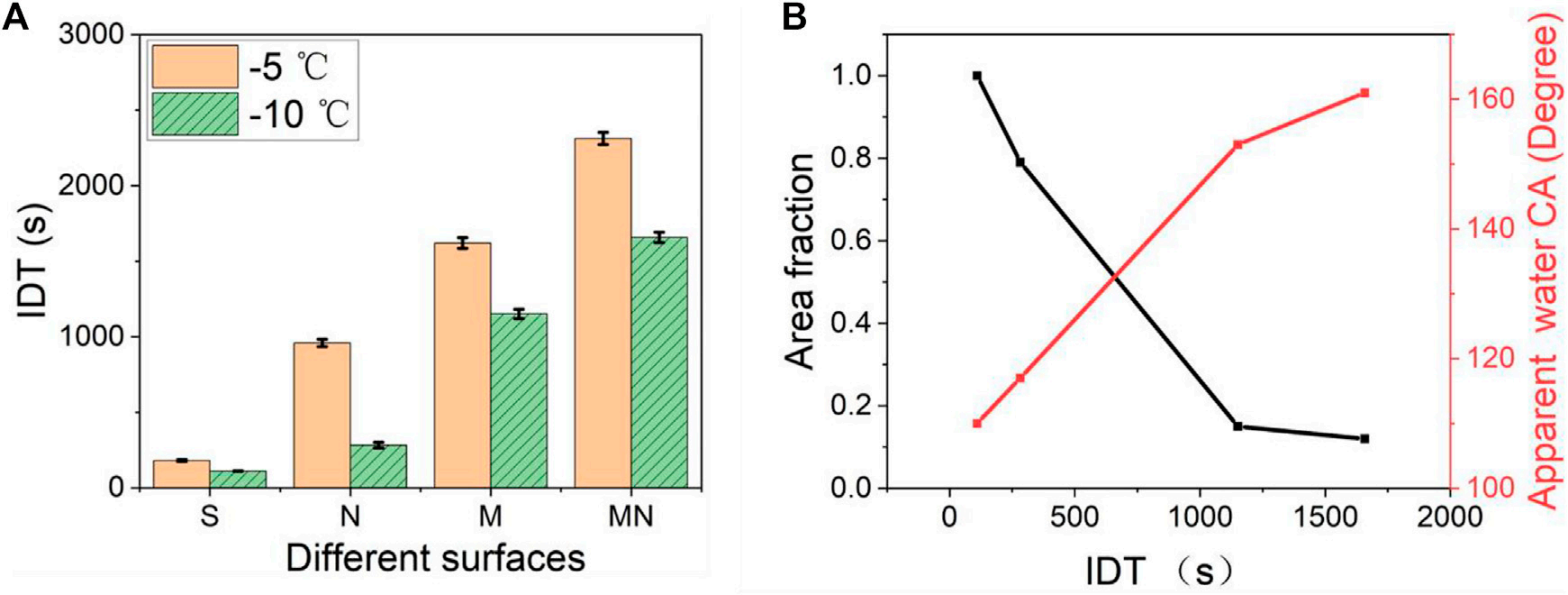
**(A)** Shows the contrast on the IDT of the various structured surfaces at −5°C and −10°C. The MN surface shows the longest DT of 2313 ± 40 s while the S surface shows the shortest IDT of 180 ± 6 s **(B)** Shows the relationship between the area fraction, apparent water contact angle, and the IDT at −10°. The apparent water contact angle is the sum of all the contributions of the liquid-solid and liquid-vapor interfaces as expressed in the Cassie–Baxter equation which is obtained using the contact angle goniometer and the ImageJ software.

Figure 8A shows the statistical contrasting column plots of the IDT of the various surfaces at −5°C and −10°C. Supplementary Video S2 recorded the whole process of water droplets’ transition from the liquid state to the ice state for the S and MN surfaces. The high IDT values obtained from the superhydrophobic surfaces can be attributed to the fact that heat transfer proceeds majorly over the contact area between ice and the structured pillars. Since the water droplet sits atop the air pockets on the superhydrophobic surfaces, and due to the low thermal conductivity of air, the structured pillars served as the primary mode of heat transfer in the vertical direction between the cold silicon substrate and the water droplet. The pockets of air between neighboring structured pillars and the water droplet interface act as a thermal absorbing layer (heat block) leading to decreased heat transfer efficiency. Furthermore, the classical nucleation theory suggests that the larger the CA of the substrate, the greater the free-energy barrier required for the ice nucleus formation, and the smaller the rate of nucleation, making the ice formation more difficult and slower (Varanasi et al., 2009; Varanasi et al., 2010). As seen in Figure 8B, the smaller the apparent contact area, the lower the heat transfer efficiency resulting in increased IDT. MN surface which has the largest CA with the least area fraction showed the longest IDT signifying excellent anti-icing behavior. Similar observations have been reported elsewhere (Li and Guo, 2018; Nguyen et al., 2018). In practice, a reduced ice nucleation temperature will enable more equipment and devices to be safely and effectively employed in environments of lower temperatures thereby generating greater productivity and profitability. Likewise, a longer IDT will increase the chances of water removal from the surface before the ice formation.

To further increase the anti-ice properties, both microstructures and nanostructures can be optimized. The micropillar gap, area fractions, heights can be easily defined by the UV-lithography and the DRIE etching step. The nanowire heights, material type can be optimized in the GLAD step by changing the deposition time and using more thermally insulative target materials such as titanium oxide (TiO2) (Fresno et al., 2021) and tin oxide (SnO2) (Chetri and Dhar, 2019). The gap of the GLAD nanowires is more difficult to change mostly determined by the initial nucleation but can be controlled in certain degrees using a prefabricated seed layer (Dick et al., 2003). The advantages of our fabrication approach are the scalability and cost-effectiveness, essential for the application of anti-ice applications.

## Conclusion

A new scalable and cost-effective method of fabricating hierarchical MN surfaces consisting of a standard microfabrication process and GLAD was demonstrated. The exciting advantage of GLAD lies in its versatility in the control and design of different types of nanostructures. The wettability analysis test results show that the contact angle of liquid droplets depends on the area fraction and is not affected by the shape of the microstructure (M surface). The obtained hierarchical MN surface offered a large water contact angle of ∼161° and sliding angle of <10° indicating good self-cleaning potentials. In addition, the surface showed an excellent icing delay time of 2313 s and 1658 s at −5°C and −10°C respectively, both are more than 10 times the icing delay time of smooth surfaces. This high icing delay time was attributed to the larger CA of the surface contributing to a higher energy barrier for ice nucleation. Besides, the CA and the IDT of the three different MN surfaces showed good consistency due to the standardization of the fabrication process.

## Data Availability

The raw data supporting the conclusion of this article will be made available by the authors, without undue reservation.
