# Supplementary material for: Bio-Inspired Hierarchical Micro/Nanostructured Surfaces for Superhydrophobic and Anti-Ice Applications
Source: Front Bioeng Biotechnol. 2022 Mar 21;10:872268. doi: 10.3389/fbioe.2022.872268 (PMC8977784; doi:10.3389/fbioe.2022.872268)
Supplement: Supplementary file 1 [file DataSheet1.pdf]

Bio-inspired Hierarchical micro/nanostructured surfaces for superhydrophobic and anti-ice applications

Lansheng Zhang[1†], Paul C. Uzoma[1†], Chu Xiaoyang[1], Oleksiy V. Penkov[1], Huan Hu[*1,2]

[1]ZJU-UIUC Institute, International Campus, Zhejiang University, Haining 314400, China.

[2]State Key laboratory of Fluidic Power & Mechanical Systems, Zhejiang University, Hangzhou, China

† These authors have contributed equally to this work and share the first authorship

*** Correspondence:**
Corresponding Author
huanhu@intl.zju.edu.cn

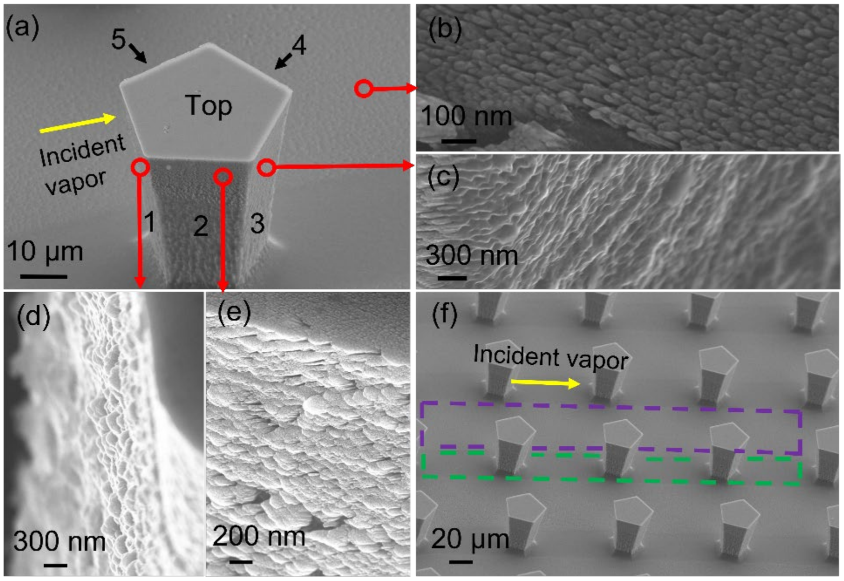

Figure S1. The SEM pictures showing the deposition of the nanowires on the different sections of the micropillars.

Nanowires were deposited on some sidewalls of the micropillars and the bottom surface as described in Figure S1. The key factors to have the nanowires on surfaces are that the incident evaporation vapor is not blocked by other micropillars and the incident evaporation vapor has a certain angle with the surface similarly like with the top surfaces.

In Figure S1, the incident vapor and sidewall 1 are perpendicular, so there are no nanowires on sidewall 1 (as seen in (d)). The SEM images show that there are nanowires on the sidewalls 2 (as seen in (e)). However, these nanowires are different from the ones observed at the top surface which is denser because of the deposition angle differences. Sidewall 5 has the same nanowires as sidewall 2 because these two surfaces are symmetrical regarding to the incident vapor direction as indicated by the yellow arrow. Sidewalls 3 and 4 are the backsides of the micropillars and are somewhat shielded from the incident vapor, so there are no nanowires deposited on them. They are shown as rough surface sidewalls obtained through the DRIE process, as seen in figure 1 (c). These rough surfaces can also enhance the hydrophobic properties.

Figure 1 (b) shows the nanowires on the bottom surface which are the same as the nanowires on the top surface. But the bottom surfaces in the shadow area of the evaporation vapors have no nanowires because the vapors are blocked by the micropillars. For example, the purple area shown in figure 1 (f) has the nanowires but the green area does not have nanowires because the incident vapor was shadowed by the micropillars.

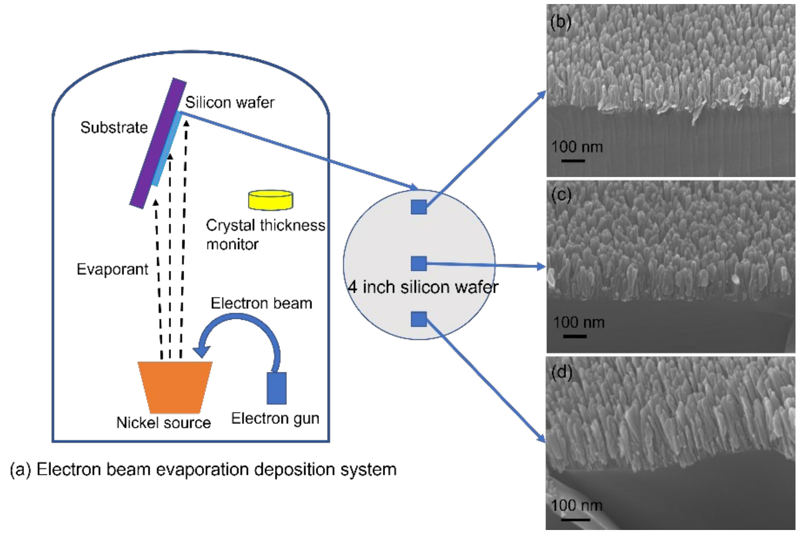

Figure S2. Schematic illustration of effects of the size and position of the substrate on the size of the nanowires. The farther the surface of the substrate is from the evaporant, the smaller the length of the nanowires. (b) length scale ranges between 90nm-110nm, (c) length scale ranges between 130nm-140nm, (d) length scale ranges between 160nm-180nm)

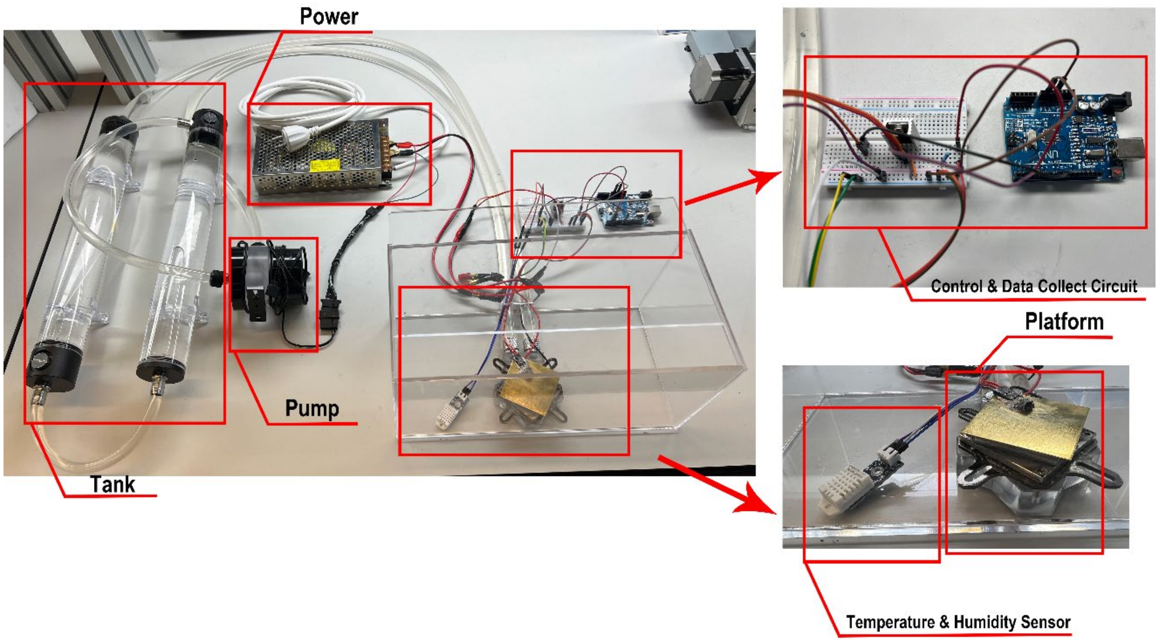

Figure S3. The anti-icing setup for measuring the ice delay time (IDT).

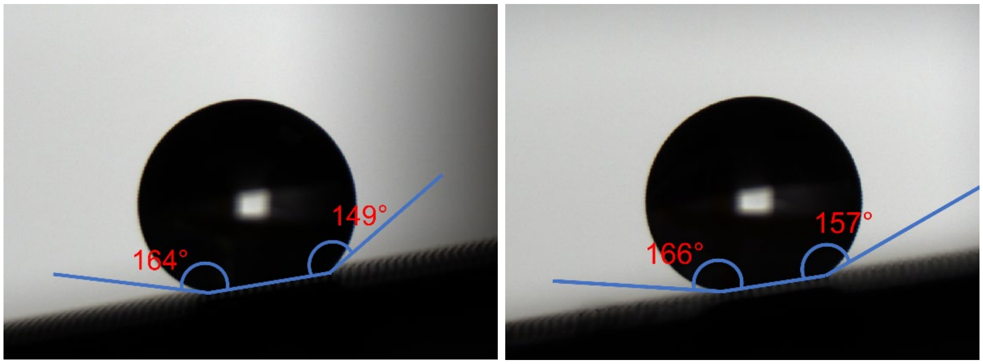

M MN

Figure S4. Advancing and receding CAs of water droplets on M and MN surfaces.

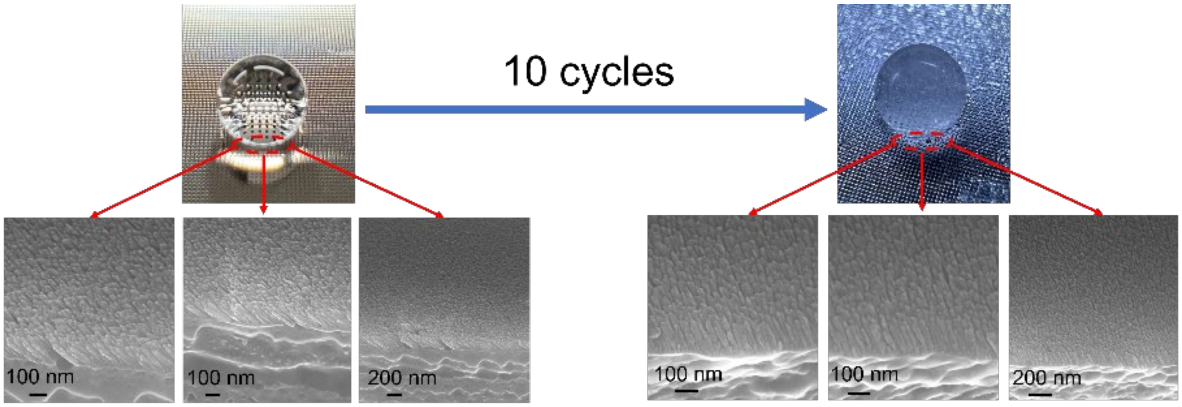

Figure S5. SEM images of the MN surfaces after 10 cycles of icing/deicing. There is no obvious damage on the MN surfaces indicating that the nanopillars were not destroyed by the ice formation.

Video S1- Sliding angle.mp4

Video S2-Ice delay time at 10 degrees.mp4
